# Cod Liver Oil, but Not Retinoic Acid, Treatment Restores Bone Thickness in a Vitamin A-Deficient Rat

**DOI:** 10.3390/nu14030486

**Published:** 2022-01-22

**Authors:** Richard C. Baybutt, Joseph T. Standard, Daniel Dim, Tim Quinn, Hana Hamdan, Dingbo Lin, Kyle Kunz, Zachary S. Bomstein, Benjamin J. Estorge, Betty Herndon, Hamid Zia, Ahmad Mansour, Manesha Lankachandra, Agostino Molteni

**Affiliations:** 1 Department of Applied Health Science, Wheaton College, Wheaton, IL 60187, USA; joestandard@gmail.com (J.T.S.); kunz.kyle@gmail.com (K.K.); 2 Department of Nutrition Science, East Carolina University, Greenville, NC 27834, USA; zsbomstein@uncg.edu (Z.S.B.); estorgeb17@students.ecu.edu (B.J.E.); 3 Department of Pathology and Pharmacology, UMKC School of Medicine, Kansas City, MO 64110, USA; dimd@umkc.edu (D.D.); quinnt@umkc.edu (T.Q.); hamdanh@umkc.edu (H.H.); herndonb@umkc.edu (B.H.); ziah@umkc.edu (H.Z.); mansoura@umkc.edu (A.M.); moltenia@umkc.edu (A.M.); 4 Department of Nutritional Sciences, Oklahoma State University, Stillwater, OK 74078, USA; dingbo.lin@okstate.edu; 5 Englewood Orthopedic Associates, 410 South Van Brunt Street, Englewood, NJ 07631, USA; klankachandram@umkc.edu

**Keywords:** vitamin A deficiency, retinoic acid, retinol, rats, osteoblast

## Abstract

Vitamin A plays a prominent role for maintaining optimal bone status, but its impact upon the bone in response to vitamin A deficiency is not well defined. The purpose of this study was to evaluate how replenishing vitamin A by either whole food cod liver oil (COD) or the active metabolite of vitamin A, retinoic acid (RA), altered bone thickness of vitamin A-deficient (VAD) rats. Weanling rats were administered a control diet (CTRL) or VAD diet for 9 weeks. This was followed by four weeks of treatment in which the VAD group was divided into the following 4 subgroups: (1) VAD (9 weeks)-VAD (4 weeks); (2) VAD-CTRL; (3) VAD-COD; and (4) VAD-RA. Compared to controls, VAD rats had thicker bones which showed marked dysplasia. VAD-rats treated with COD produced a thinner bone that was not significantly different from that of untreated rats. In contrast, RA did not significantly change the thicker bone, and also had significantly greater periosteal and endosteal osteoblast numbers compared to VAD-COD. Active osteoclasts were not detected in VAD rats, nor during the treatment period. These findings suggest that the abnormal bone thickness in VAD rats appears to be more effectively restored to bone thickness of untreated control rats when treated with COD.

## 1. Introduction

Bone growth and development is a process that requires the continual equilibrium of bone resorption through osteoclasts and bone deposition through osteoblast. The osteoclasts cause the bone tissue to release its calcium into systemic circulation [1], while osteoblast activation causes bone tissue to take up calcium via secretions from the osteoid portion of the bone [2]. Calcium and phosphorus are key building materials of bone that are impacted by hormones such as parathyroid hormone (PTH), in addition to micronutrients such as vitamin A, vitamin D, vitamin C and vitamin K [3]. While the role of calcium and vitamin D have been reasonably well defined in bone development, the role of vitamin A is not well understood. Many studies have reported that vitamin A excess is a potential risk factor for initiating bone loss. In a case-control study of 247 women aged 40–76, it was found that individuals with intakes of dietary retinol greater than 1.5 g/day had significantly reduced Bone Mineral Density (BMD) in several different bones, compared to individuals who consumed less than 0.5 g/day [4]. Vitamin A and vitamin D play important roles in maintaining proper bone structure through activating osteoclasts and osteoblasts, respectively, in the continual bone degradation and formation process. This continual renewal of bone structure allows for bone to adapt to stressors that are placed upon it and for bone growth.

Various bioactive components have been found to promote the activity of osteoclasts or osteoblasts which can cause the equilibrium to shift from net bone loss to bone growth [5]. Vitamin A, in the form of all-trans-retinoic acid, has been found to directly promote bone resorption, which leads to decreased bone mineral density (BMD) in rats independent of cholecalciferol (vitamin D_3_), calcium or phosphorus levels [6]. All-trans-retinoic acid has also been found to stimulate mature isolated osteoclasts through increased gene expression of cathepsin K/osteoclast-2 (OC-2) and increased expression of retinoic acid receptor α mRNA and retinoid X-receptor β mRNA [7]. 

At high doses, vitamin A is the only known molecule to induce spontaneous fractures in laboratory animals, and excessive intake of vitamin A has been identified as a risk factor for fractures in humans [8]. Previous studies show that excess Vitamin A or Vitamin A toxicity has been associated with osteopenia, fractures, deformities and growth arrest [9,10,11]. Rats receiving excess vitamin A treatment had increased bone resorption, osteoclastosis, and a paucity of trabecular surfaces covered with osteoid and lesions which were all attributed to hypervitaminosis A [9]. At the same time, the levels of other compounds promoting bone resorption, such as PTH, 1,25-dihydroxyvitamin D and 25-hydroxyvitamin D in the vitamin A excess-treated rats, were similar to the levels of the control animals, suggesting that the increased bone resorption must be due to the increased dosage of vitamin A, independently of these other bone-related, bio-active compounds [9]. These studies demonstrate that high dosages of vitamin A are involved with a significant decrease in bone mineral content (BMC), and eventually BMD of the bone tissue [12]. 

Little is known about bone health in response to vitamin A deficiency. Mellanby et al. showed that VAD (vitamin A-deficient) induced thickened bone in a dog model, attributing the excess bone growth to increased osteoblast activity [13]. The purpose of this study was to use a VAD rat model to evaluate how replenishing vitamin A by either the whole food cod liver oil or vitamin A’s active metabolite RA altered bone status and affected osteoclast and osteoblast numbers. Based on Mellanby’s work [13], we predicted that the VAD group would have a thicker bone. As suggested by Mellanby et al., the thicker bone may be due to an increase in osteoblast activity; however, it may also be due to a decrease in osteoclast activity. After we evaluated the impact of VAD on bone status, we replenished vitamin A in the form of the whole food cod liver oil or its active metabolite retinoic acid, to determine which form was most effective in restoring the bone. 

## 2. Materials and Methods

### 2.1. Animals

Forty-four male Charles River Sprague-Dawley rats (Indianapolis, IN, USA) were individually housed in stainless steel cages at 24 ± 1 °C with a 12-hour light-dark cycle with humidity control. At the start of the experiment, each of the rats weighed 50–60 g. Animal care and use were approved by the Institutional Animal Care and Use Committee (IACUC#F09-006A and F09-006B, approved 13 August 2009) of Wheaton College. 

### 2.2. Diet

An overview of dietary treatment groupings is shown in Table 1. Rats were briefly randomly assigned to two initial groups: control and VAD diets. After 9 weeks on these diets, 12 rats (6 VAD, 6 CTRL) were administered sodium pentobarbital intraperitoneally. Femur tissue was collected and fixed in formalin 10% for histological studies, and the rats were euthanized. For weeks 10–13, the remaining control rats remained on a control diet (CTRL-CTRL). Meanwhile, the VAD rats were divided into four dietary groups: (1) Vitamin A deficient diet (VAD-VAD); (2) control diet (VAD-CTRL); (3) control diet + cod liver oil (VAD-COD); and (4) control diet + retinoic acid (VAD-RA). The vitamin A was provided in two different forms as a whole food as cod liver oil (COD), or in its active metabolite form of retinoic acid (RA). The COD contained ~5.7 mg/kg diet retinol and a RA diet containing ~13.3 mg/kg diet of vitamin A. The Vitamin A in cod liver oil was chosen as a dietary source of vitamin A, as opposed to the supplemental form in the retinoic acid treatment group based on a previous study [12]. The rats were fed an ad libitum diet and had ad libitum tap drinking water available. Daily food intake and weekly rat body weights were recorded. 

### 2.3. Bone Analysis

**Figure 1:**
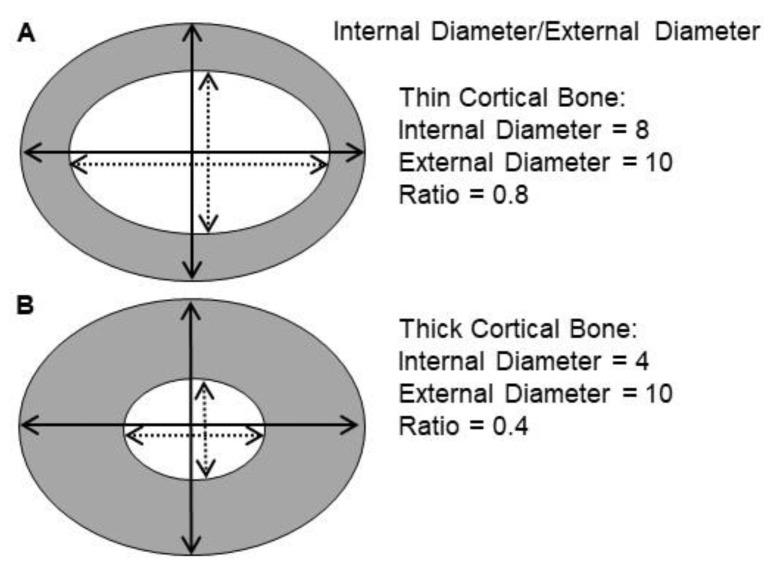
Example of ratio of internal to external diameter measurements conducted at cross-sections of femur in rats. Cortical bone is colored gray, while trabecular bone is colored white. Thinner cortical bone (**A**) has a higher internal/external ratio (0.8) compared to a thicker cortical bone (**B**) that has a lower internal/external ratio (0.4). Note picture is not drawn to scale.

At the end of the 9-week VAD diet and at the end or the 4-week vitamin A replenishment treatment, respectively, the rats were given an inhaled anesthetic (Isofluorane, Abbott Labs, Chicago, IL, USA) to prevent movement while placed on a scanning bed. While on the bed, bone scans were performed via Dual-emission X-Ray absorptiometry (DEXA) (GE, Fairfield, CT, USA) to determine BMD and bone mineral content BMC. Following DEXA analysis, the rats were euthanized, the right femurs were collected and fixed with 10% formalin and sent to pathologists for histological studies. After decalcification and staining by H&E, cross-sectional internal and external diameters were averaged at the widest and narrowest points of the femur. The ratio of internal to external diameter of bone was measured, with an inverse relationship between the ratio value and cortical bone thickness. An example is shown of how the ratio value is inversely related to cortical bone thickness (Figure 1). 

### 2.4. Osteoblast Cell Count & Osteoclast Cell Count

Osteoblast counting was performed on the femur sections after decalcification, staining by H&E and evaluated by light microscopy at 400× by three pathologists independently (D.V., H.Z. and A.M.), and being unaware of slide identification. The three counts were then summed and averaged. A CD68 immunohistochemical stain using an immunoperoxidase technique was performed on all bone sections to confirm the count of osteoblasts. Osteoclast counting was conducted in the same way. 

### 2.5. Statistical Analysis

Data were expressed as mean +/− standard error of the mean (SEM). The five groups were compared using a one-way ANOVA using IBM SPSS Software, Chicago, IL, USA. A least square difference (LSD) post-hoc test was used to detect significance following one-way ANOVA. Graphs were formulated using statistical software and Microsoft Office.

## 3. Results

### 3.1. Mortality

No rats died from dietary treatment during the thirteen-week study. Two rats died due to overexposure to anesthesia when conducting the bone readings using the DEXA. Twelve rats were euthanized at week 9 to examine the impact of VAD rats on bone status in the VAD group (*n* = 6) and the CTRL group (*n* = 6).

### 3.2. Food Intake and Body Weight Gain

**Figure 2:**
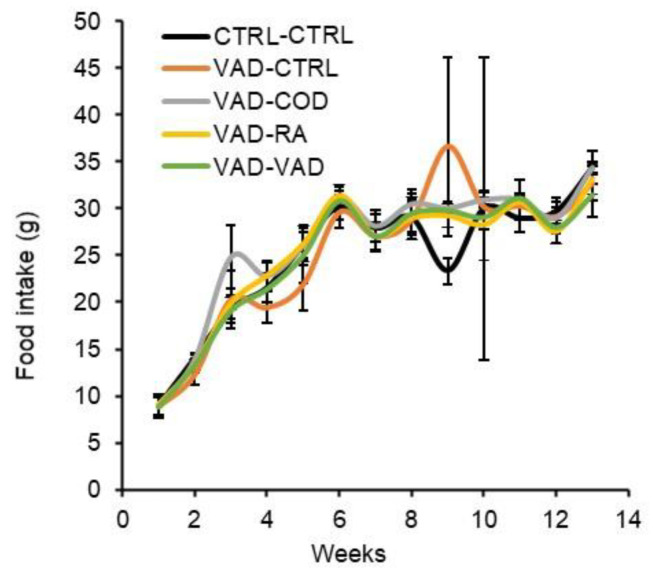
Weekly average food intakes are given as mean ± standard error (*p* < 0.05). There was no significant difference in the average food intake among the different groups. (CTRL: control diet, VAD: vitamin A deficient diet, COD: cod liver oil diet, RA: retinoic acid diet).

**Figure 3:**
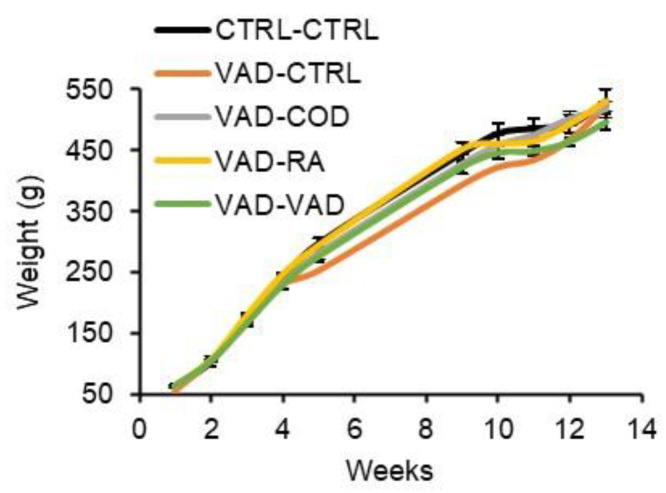
Weekly average body weights of the groups are given as mean ± standard error. There were no statistically significant differences in weight gain among the groups at *p* < 0.05, except at week 12 when the control group weighed significantly more than the VAD-VAD group. (CTRL: control diet, VAD: vitamin A deficient diet, COD: cod liver oil diet, RA: retinoic acid diet).

The weekly average food intake for the rats is reported in Figure 2. Both the control and VAD rats consumed food at similar rates. The body weights followed a similar trend to the food consumption rates, and are shown in Figure 3. At week 13, the end of the study, the average body weight was not significantly different among any of the different groups. 

### 3.3. DEXA

Using a student t-test, no significant difference was detected between VAD versus the control at week 9 in BMC (*p* = 0.52), although there appeared to be a trend of an increase in BMD in the VAD rats (*p* = 0.07). After four weeks of retinoid treatment, ANOVA analysis did not show statistical significance for BMD or BMC when comparing amongst the groups of CTRL-CTRL, VAD-CTRL, VAD-COD, VAD-RA and VAD-VAD (data not shown). 

### 3.4. Bone Histopathology

**Figure 4:**
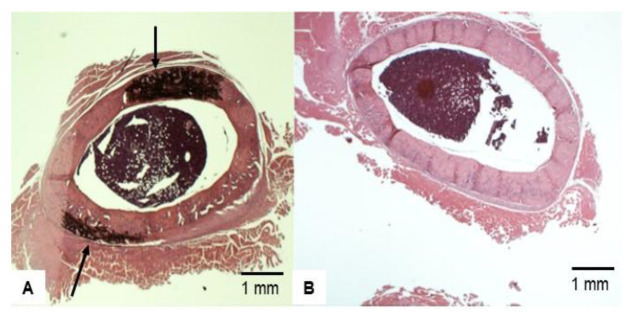
Representative H&E staining sample of cross-sectional view of femoral bone after 9 weeks treatment: (**A**) VAD and (**B**) Control. Rats fed a vitamin A-deficient diet had a lower ratio of internal bone vs. the external (compact) bone (i.e., a thicker bone), with marked dysplasia (Arrowed in (**A**)) relative to the bone of rats on a normal diet (**B**).

**Figure 5:**
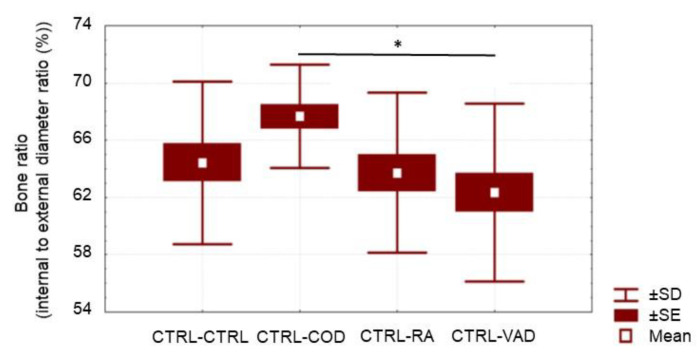
Measurement of bone thickness via ratio of internal/external bone diameter amongst the four treated VAD groups, with a higher ratio value indicating reduced cortical bone thickness. The rats were on a VAD diet for 9 weeks, and then were given four different treatments for 4 more weeks: CTRL, COD, RA and VAD. Please note the higher internal/external bone diameter ratios for the COD treatment indicating a significantly thinner bone compared to the thicker bone of the VAD group throughout the study (* Statistical difference, *p* = 0.016), but the bones of the COD treated rats were not significantly different from the rats remaining on the control diet throughout the study, 67.2 ± 3.5 for CTRL-CTRL (mean ± SE, data not depicted in figure) compared to 67.9 ± 2.1 for VAD-COD. The bones of the RA-treated rats had a smaller internal to external bone diameter ratio, indicating a thicker bone that was not significantly different from the VAD-VAD group.

As demonstrated in Figure 1, a thicker cortical bone has a lower ratio of internal/external diameter value, showing an inverse relationship between the ratio value and cortical bone thickness. Representative femur cross-sections of VAD versus control are shown at 9 weeks (Figure 4). Rats fed a vitamin A-deficient diet had a lower ratio of internal bone vs. the external (compact) bone (i.e., a thicker bone), with marked dysplasia (Figure 4A) relative to the bone of “rats” on a normal diet (Figure 4B). After the rats were on a VAD diet for 9 weeks, they were given four different treatments for 4 more weeks: (a) VAD-CTRL; (b) VAD-COD; (c) VAD-RA; and (d) VAD-VAD. The bone thickness for each respective group was determined by the ratio of the internal/external bone diameter. Cortical bone thickness of the four respective treatment groups (Figure 5) indicated that VAD-COD had significantly thinner cortical bone compared to the rats that remained on the vitamin A-deficient diet (VAD-COD compared to VAD-VAD, *p* = 0.016), and the bone thickness of the COD treated rats was not significantly different than the untreated control rats (67.2 ± 3.5 for control (mean ± SE) compared to 67.9 ± 2.1 for COD treated), suggesting a restoration by COD. In contrast, the bones of the VAD rats supplemented with RA were thicker than the COD-treated rats, and not significantly different from the rats remaining on the VAD diet throughout the study, suggesting there was no change in response to RA treatment. 

### 3.5. Osteoblast and Osteoclast Cell Count

**Figure 6:**
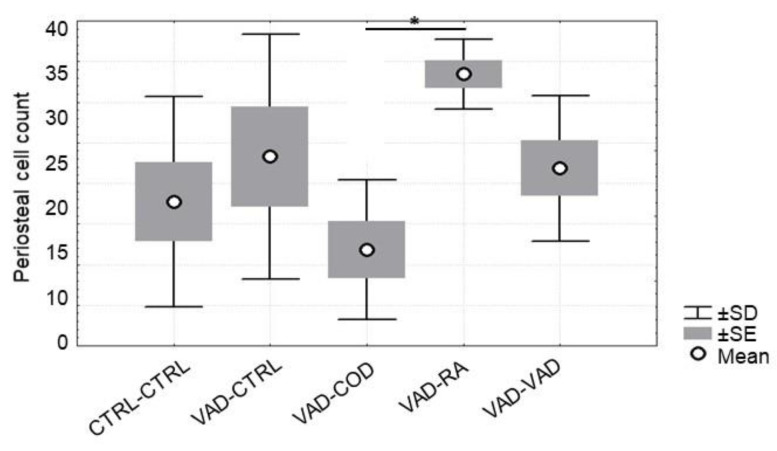
Average osteoblast cell counts for periosteal bone at 13 weeks. The rats were on a VAD diet for 9 weeks, and were then given four different treatments for 4 more weeks: CTRL-CTRL; VAD-CTRL; VAD-COD; VAD-RA; and VAD-VAD. The increased osteoblast counts for the VAD-RA group were significantly greater than the number of osteoblasts for the VAD-COD group (* Statistical difference, *p* < 0.01).

**Figure 7:**
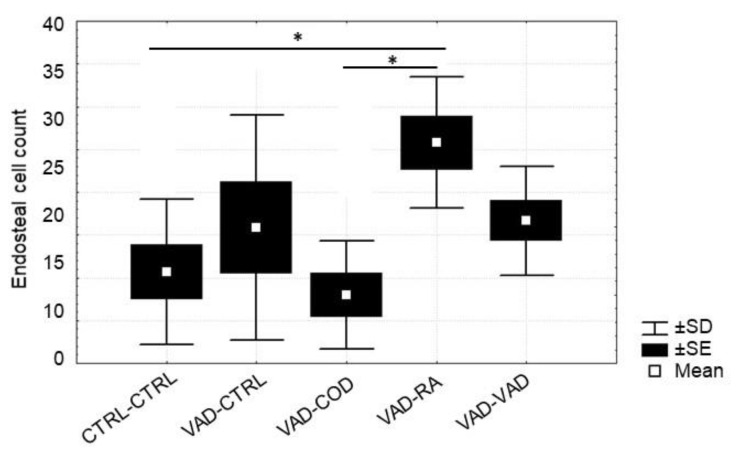
Average osteoblast cell counts for endosteal bone at 13 weeks. The rats were on a VAD diet for 9 weeks, and were then given four different treatments for 4 more weeks: CTRL-CTRL; VAD-CTRL; VAD-COD; VAD-RA; and VAD-VAD. The pattern of osteoblast cell counts for endosteal bone was similar to that of the periosteal bone, with a significant increased count for the VAD-RA group compared to the CTRL-CTRL group (* Statistical difference, *p* = 0.03), and compared to the VAD-COD group (* Statistical difference, *p* = 0.01).

The effect of the different forms of vitamin A on osteoblast counts in both the perios-teal bone (Figure 6) and the endosteal bone (Figure 7) were measured and averaged for each experimental and control group for week 13. For periosteal bone, there were significantly more osteoblasts present for the VAD-RA group when compared to the VAD-COD group (*p* = 0.01). This trend was also observed for the endosteal bone. In addition, the VAD-RA group had significantly more osteoblasts than either the CTRL-CTRL group (*p* = 0.03) or the VAD-COD group (*p* = 0.01). Osteoclasts were not detected in the histological evaluation of the rats on the VAD diet for nine weeks, nor for any of the treatment groups at week 13. 

### 3.6. Representative Femoral Bone Cross-Section of Treated VAD Rats

**Figure 8:**
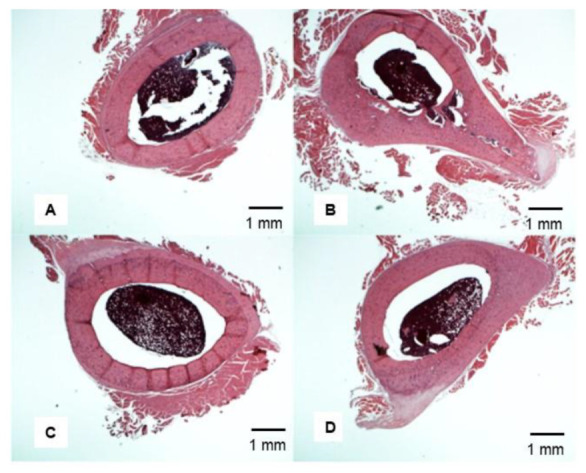
Representative H&E staining sample of cross-sectional view of femoral bone at 13 weeks. The rats were on a VAD diet for 9 weeks and then were given four different treatments for 4 more weeks: (**A**) VAD-VAD; (**B**) CTRL-CTRL; (**C**) VAD-COD; and (**D**) VAD-RA. The VAD-COD group has the thin cortical bone (**C**) and the VAD-VAD had the thick cortical bone (**D**).

A representative sample of cross-sectional views of femoral bones of rats that were on a VAD diet for nine weeks and then treated with either COD or RA. The groups are as follows: (A) VAD-VAD; (B) CTRL-CTRL; (C) VAD-COD; and (D) VAD-RA (Figure 8).

## 4. Discussion

The purpose of this study was to use a VAD rat model to evaluate how replenishing vitamin A by either the whole food cod liver oil or its active metabolite RA altered bone thickness, and also affected osteoclast and osteoblast numbers. After 9 weeks of consuming a vitamin A-deficient diet, bone morphology in rats significantly changed, producing a thicker yet dysplastic bone. When the VAD rats were treated with cod liver oil for four weeks, the femur exhibited significantly thinner cortical bone than that of the rats, which remained VAD throughout the study, and were not significantly different from untreated controls, suggesting that the COD restored the bone of deficient rats. In contrast, the thicker cortical bone of the VAD rats treated for four weeks with retinoic acid was not significantly different than that of the VAD-treated rats, which remained on the deficient diet throughout the study, and the RA-treated rats had a significantly greater number of periosteal and endosteal osteoblast than the rats on the COD diet. 

### 4.1. VAD Induces Thicker Bone

To our knowledge, this is the first time demonstrating that rats fed a VAD diet for nine weeks have their femur thickness and density increase. These effects were also reported in dogs administered a VAD diet [13,14]. The VAD-induced thicker bone may be related to osteoblast- and osteoclast-mediated interactions of RANK (Receptor Activator of Nuclear Factor Kb), RANKL (Receptor Activator of Nuclear Factor Kb Ligand) and OPG (Osteoprotegerin), the RANK-RANKL-OPG system [15]. Osteoblasts produce RANKL, which binds to RANK, a transmembrane receptor found on osteoclast precursor cells and osteocytes. Binding of RANKL to RANK induces downstream transcription of proteins promoting osteoclastogenesis [16]. On the other hand, OPG, also produced by osteoblasts, functions as a decoy receptor for RANKL, preventing its attachment to RANK, and thus reducing downstream osteoclastogenesis activities. Retinoic acid has been shown to increase osteoclast activity while decreasing osteoblast activity, a mechanism thought to be mediated through an RA-induced increase in RANKL, while decreasing OPG [15]. Collectively, the RANK-RANKL-OPG system plays an important role in bone homeostasis, and an RA-induced RANKL increase in the presence of static levels of OPG stimulates increased osteoclast activity leading to increased bone resorption [15,16,17]. Thus, without RA as in the VAD diet, there would be reduced osteoclast activity and potentially increased osteoblast activity. This corroborates our finding of no detectable active osteoclasts present within the femur of rats on a VAD diet. Further studies will be important to explore this RANK-RANKL-OPG system. 

In addition to the action of RA on osteoclast activity, the retinoid affects osteoblast activity through the inhibition of the transcription factor Runx2. Osteoblasts are derived from mesenchymal stem cells, and the osteoblast-specific commitment of these stem cells is regulated by the transcription factor Runx2 [18]. Runx2 interacts with Smad1 and Smad5 proteins, causing downstream expression of osteoblastic genes, including type 1 collagen (COL-1), bone sialoprotein (BSP), osteocalcin (OCN) and osteopontin (OPN), while also furthering the commitment of mesenchymal stem cells to an osteoblast-specific lineage [18,19]. Interestingly, retinoic acid inhibits Runx2 both in vivo and in vitro, leading to an overall inhibition of osteoblastic activity [20]. Collectively, it seems logical to infer that the thicker bone observed in VAD rats after nine weeks was likely due to a reduction in RA-stimulated osteoclast activity.

### 4.2. Cod Liver Oil-Containing Retinol Reduces External Bone Thickness after VAD

We observed that VAD rats treated with cod liver oil for four weeks exhibited a significantly thinner cortical bone than rats which remained deficient for the remainder of the treatment period, and were not significantly different from rats fed a control diet throughout the study. It is interesting to note that we did not detect active osteoclasts at the end of any of the four-week treatments. It may be that it takes longer to fully activate the osteoclasts so that they could be detected. Nevertheless, based on our finding of a bone thinning response to COD diet, it seems that there was some osteoclast activity, although not identified by our pathologists. Vitamin A in large amounts reduces cortical bone thickness through osteoclast-mediated effects on bone resorption [21]. Furthermore, after 8 days of high dietary intake of retinol, young rats exhibited significantly weaker femoral bone strength and decreased cortical bone area [8]. The authors of this study attributed this effect to osteoclast migration from the endosteum to the periosteal bone surface. We have previously demonstrated that vitamin A-induced resorption initially occurs in the periosteal bone, and subsequently in the endosteal bone [12]. In addition to decreased periosteal bone thickness, retinol has also been shown to reduce mechanical loading-induced bone formation in mice after 6-weeks of treatment, largely through decreasing the bone formation rate (BFR) of osteoblasts [22]. In future studies, it will be important to extend the treatment period to determine whether the osteoclasts would be detectable in response to COD treatment. 

This is the first time reported that VAD rats treated with the COD diet, but not with RA, more closely resembled a normal bone thickness, with a cortical bone thickness similar to that of the untreated control group. This result may be due to differences in absorption and metabolism of retinol versus retinoic acid. Retinol, after being absorbed and converted into a retinyl ester, is packaged into a chylomicron, which enters the lymphatic system, eventually making its way into the blood to target tissues before reaching the liver [23,24]. Bones are one of the primary target organs of chylomicron retinol delivery, specifically targeting osteoblasts [25]. Within the osteoblast, the delivered retinol could be converted to RA and inhibit the activation of osteoblasts and/or promote osteoclastogenesis. In contrast, dietary retinoic acid after enterocyte absorption enters the portal blood, where it is taken up and some catabolized by cyp26A in the liver [26,27,28,29]. Thus, less of the retinoic acid is available to the bone for its biological effects. Therefore, the cod liver oil retinol-induced thinner bone may be due to the more effective delivery of retinol to the bone compared to retinoic acid. 

### 4.3. Osteoblast Number Increases in Response to Supplemented Retinoic Acid

The VAD rats treated with RA had significantly greater osteoblast numbers than the rats treated with COD, in both the periosteum and the endosteum. This is consistent with the thicker bone observed for the RA-treated rats compared to the COD-treated ones. As mentioned previously, this may be due to the differences in retinoid availability to the bone tissue. The increased retinol delivered to the bone tissue via the cod liver oil diet may increase the retinol content of the osteoblast and its subsequent conversion to RA, promoting osteoclastogenesis. Taken together, it is plausible that the increase in osteoblast numbers observed in VAD rats treated with retinoic acid may be due to the decreased amount of the retinoid delivered to the bone tissue, compared to those rats treated with cod liver oil. Further research is needed to explore this assertion.

## 5. Conclusions

In our study, we showed that 9 weeks of VAD caused thickening of the cortical bone in rats. We also found and for the first time reported, that treatment of VAD-rats with COD produced a significantly thinner bone than that of VAD rats, restoring the bone to normal thickness. In contrast, VAD rats treated for four weeks with RA did not reverse the VAD-induced bone thickening, and were associated with a greater number of osteoblasts in the periosteal and endosteal bone. Our results suggest that replenishing VAD rats with whole food cod liver oil appeared to be more effective in restoring the thickness of the bone of the deficient rat than supplementing with RA, the active metabolite of vitamin A.

## Figures and Tables

**Table 1:** Experimental design with diet treatments throughout the course of study.

Diet	Diet Characterization
CTRL	Rats euthanized at week 9 after fed a control diet (*n* = 6)
CTRL-CTRL	Rats were fed a control diet for 9 weeks and
	remained on the diet for weeks 10–13 (*n* = 7)
VAD	Rats euthanized at week 9 after fed VAD diet (*n* = 6)
	After rats were fed a VAD diet for 9 weeks subsequent treatments as follows:
VAD-VAD	VAD diet for weeks 10–13 (*n* = 7)
VAD-CTRL	Control diet for weeks 10–13 (*n* = 6)
VAD-COD	Control diet + cod liver oil for weeks 10–13 (*n* = 6)
VAD-RA	Control diet + retinoic acid for weeks 10–13 (*n* = 6)

## Data Availability

Data will become available upon request when the manuscript is published.

## Abbreviations

ATRA	all-trans retinoic acid
BFR	bone formation rate
BMC	bone mineral content
BMD	bone mineral density
BSP	bone sialoprotein
COD	cod liver oil
COL-1	type 1 collagen
CTRL	control
DEXA	dual-emission X-ray absorptiometry
OC-2	osteoclast-2
OCN	osteocalcin
OPG	osteoprotegerin
OPN	osteopontin
RA	retinoic acid
RANK	receptor activator of nuclear Kb
RANKL	receptor activator of nuclear Kb ligand
PTH	parathyroid hormone
VAD	vitamin A deficient

## References

[1] Boyle W.J., Simonet W.S., Lacey D.L. (2003). Osteoclast differentiation and activation. Nature.

[2] Ducy P., Schinke T., Karsenty G. (2000). The Osteoblast: A Sophisticated Fibroblast under Central Surveillance. Science.

[3] Ahmadieh H., Arabi A. (2011). Vitamins and bone health: Beyond calcium and vitamin D. Nutr. Rev..

[4] Melhus H., Michaëlsson K., Kindmark A., Bergstrom R., Holmberg L., Mallmin H., Wolk A., Ljunghall S. (1998). Excessive Dietary Intake of Vitamin A Is Associated with Reduced Bone Mineral Density and Increased Risk for Hip Fracture. Ann. Intern. Med..

[5] Morgan S.L. (2009). Nutrition and Bone: It is More than Calcium and Vitamin D. Women’s Health.

[6] Rohde C.M., DeLuca H. (2003). Bone resorption activity of all-trans retinoic acid is independent of vitamin D in rats. J. Nutr..

[7] Saneshige S., Mano H., Tezuka K., Kakudo S., Mori Y., Honda Y., Itabashi A., Yamada T., Miyata K., Hakeda Y. (1995). Retinoic acid directly stimulates osteoclastic bone resorption and gene expression of cathepsin K/OC-2. Biochem. J..

[8] Lind T., Lind P.M., Jacobson A., Hu L., Sundqvist A., Risteli J., Yebra-Rodriguez A., Rodriguez-Navarro A., Andersson G., Melhus H. (2011). High dietary intake of retinol leads to bone marrow hypoxia and diaphyseal endosteal mineralization in rats. Bone.

[9] Hough S., Avioli L.V., Muir H., Gelderblom D., Jenkins G., Kurasi H., Slatopolsky E., Bergfeld M.A., Teitelbaum S. (1988). Effects of Hypervitaminosis A on the Bone and Mineral Metabolism of the Rat*. Endocrinology.

[10] Oreffo R.O., Teti A., Triffitt J., Francis M., Carano A., Zallone Z.A. (2009). Effect of vitamin a on bone resorption: Evidence for direct stimulation of isolated chicken osteoclasts by retinol and retinoic acid. J. Bone Miner. Res..

[11] Wu A.-M., Huang C.-Q., Lin Z.-K., Tian N.-F., Ni W.-F., Wang X.-Y., Xu H.-Z., Chi Y.-L. (2014). The Relationship Between Vitamin A and Risk of Fracture: Meta-Analysis of Prospective Studies. J. Bone Miner. Res..

[12] Xue Y., Haub M.D., Smith B.W., Baybutt R.C. (2011). Decreases in Bone Mineral Content by Dietary All-Trans Retinoic Acid Precede Decreases in Bone Mineral Density in a Weanling Rat Model of Cigarette Smoke—Induced Lung Injuries. Int. J. Vitam. Nutr. Res..

[13] Mellanby E. (1941). Skeletal changes affecting the nervous system produced in young dogs by diets deficient in vitamin A. J. Physiol..

[14] Mellanby E. (1947). Vitamin A and bone growth: The reversibility of vitamin A-deficiency changes. J. Physiol..

[15] Conaway H.H., Pirhayati A., Persson E., Pettersson U., Svensson O., Lindholm C., Henning P., Tuckermann J., Lerner U.H. (2011). Retinoids Stimulate Periosteal Bone Resorption by Enhancing the Protein RANKL, a Response Inhibited by Monomeric Glucocorticoid Receptor. J. Biol. Chem..

[16] Walsh M., Choi Y. (2014). Biology of the RANKL–RANK–OPG system in immunity, bone and beyond. Front. Immunol..

[17] Kühn M.C., Willenberg H.S., Schott M., Papewalis C., Stumpf U., Flohe S., Scherbaum W.A., Schinner S. (2012). Adipocyte-secreted factors increase osteoblast proliferation and the OPG/RANKL ratio to influence osteoclast formation. Mol. Cell. Endocrinol..

[18] Lin G.L., Hankenson K.D. (2011). Integration of BMP, Wnt, and notch signaling pathways in osteoblast differentiation. J. Cell. Biochem..

[19] Nishimura R., Hata K., Ikeda F., Ichida F., Shimoyama A., Matsubara T., Wada M., Amano K., Yoneda T. (2008). Signal transduction and transcriptional regulation during mesenchymal cell differentiation. J. Bone Miner. Metab..

[20] Lind T., Sundqvist A., Hu L., Pejler G., Andersson G., Jacobson A., Melhus H. (2013). Vitamin A Is a Negative Regulator of Osteoblast Mineralization. PLoS ONE.

[21] Lionikaite V., Gustafsson K.L., Westerlund A., Windahl S.H., Koskela A., Tuukkanen J., Johansson H., Ohlsson C., Conaway H.H., Henning P. (2018). Clinically relevant doses of vitamin A decrease cortical bone mass in mice. J. Endocrinol..

[22] Lionikaite V., Henning P., Drevinge C., Shah F.A., Palmquist A., Wikström P., Windahl S.H., Lerner U.H. (2019). Vitamin A decreases the anabolic bone response to mechanical loading by suppressing bone formation. FASEB J..

[23] Harrison E.H. (2012). Mechanisms involved in the intestinal absorption of dietary vitamin A and provitamin A carotenoids. Biochim. et Biophys. Acta (BBA)-Mol. Cell Biol. Lipids.

[24] Schreiber R., Taschler U., Preiss-Landl K., Wongsiriroj N., Zimmermann R., Lass A. (2012). Retinyl ester hydrolases and their roles in vitamin A homeostasis. Biochim. et Biophys. Acta (BBA)-Mol. Cell Biol. Lipids.

[25] Niemeier A., Niedzielska D., Secer R., Schilling A., Merkel M., Enrich C., Rensen P.C., Heeren J. (2008). Uptake of postprandial lipoproteins into bone in vivo: Impact on osteoblast function. Bone.

[26] Fidge N.H., Shiratori T., Ganguly J., Goodman D.S. (1968). Pathways of absorption of retinal and retinoic acid in the rat. J. Lipid Res..

[27] Saadeddin A., Torres-Molina F., Cárcel-Trullols J., Araico A., Peris J.-E. (2004). Pharmacokinetics of the time-dependent elimination of all-trans-retinoic acid in rats. AAPS PharmSci.

[28] Muindi J., Frankel S.R., Miller W.H., Jakubowski A., Scheinberg D.A., Young C.W., Dmitrovsky E., Warrell R.P. (1992). Continuous treatment with all-trans retinoic acid causes a progressive reduction in plasma drug concentrations: Implications for relapse and retinoid “resistance” in patients with acute promyelocytic leukemia. Blood.

[29] Jing J., Nelson C., Paik J., Shirasaka Y., Amory J., Isoherranen N. (2017). Physiologically Based Pharmacokinetic Model of All-trans-Retinoic Acid with Application to Cancer Populations and Drug Interactions. J. Pharmacol. Exp. Ther..

